# Correlations between Gray Matter and White Matter Degeneration in Pure Alzheimer’s Disease, Pure Subcortical Vascular Dementia, and Mixed Dementia

**DOI:** 10.1038/s41598-017-10074-x

**Published:** 2017-08-25

**Authors:** Hyemin Jang, Hunki Kwon, Jin-Ju Yang, Jinwoo Hong, Yeshin Kim, Ko Woon Kim, Jin San Lee, Young Kyoung Jang, Sung Tae Kim, Kyung Han Lee, Jae Hong Lee, Duk L. Na, Sang Won Seo, Hee Jin Kim, Jong-Min Lee

**Affiliations:** 1 Department of Neurology, Samsung Medical Center, Sungkyunkwan University School of Medicine, Seoul, Korea; 2 Radiology Samsung Medical Center, Sungkyunkwan University School of Medicine, Seoul, Korea; 3 Nuclear Medicine, Samsung Medical Center, Sungkyunkwan University School of Medicine, Seoul, Korea; 4 Neuroscience Center, Samsung Medical Center, Seoul, Korea; 5 Department of Biomedical Engineering, Hanyang University, Seoul, Korea; 6 Department of Neurology, Chonbuk National University Hospital, Chonbuk National University Medical school, JeonJu, Korea; 7 Department of Neurology, Kyung Hee University Hospital, Seoul, Korea; 8 Department of Neurology, Asan Medical Center, Ulsan University School of Medicine, Seoul, Korea; 9 Department of Health Sciences and Technology, SAIHST, Sungkyunkwan University, Seoul, Korea; 10 Stem Cell & Regenerative Medicine Institute, Samsung Medical Center, Seoul, Korea; 11 Department of Clinical Research Design & Evaluation, SAIHST, Sungkyunkwan University, Seoul, Korea

## Abstract

Alzheimer’s disease dementia (ADD) and subcortical vascular dementia (SVaD) both show cortical thinning and white matter (WM) microstructural changes. We evaluated different patterns of correlation between gray matter (GM) and WM microstructural changes in pure ADD, pure SVaD, and mixed dementia. We enrolled 40 Pittsburgh compound B (PiB) positive ADD patients without WM hyperintensities (pure ADD), 32 PiB negative SVaD patients (pure SVaD), 23 PiB positive SVaD patients (mixed dementia), and 56 normal controls. WM microstructural integrity was quantified using fractional anisotropy (FA), axial diffusivity (DA), and radial diffusivity (DR) values. We used sparse canonical correlation analysis to show correlated regions of cortical thinning and WM microstructural changes. In pure ADD patients, lower FA in the frontoparietal area correlated with cortical thinning in the left inferior parietal lobule and bilateral paracentral lobules. In pure SVaD patients, lower FA and higher DR across extensive WM regions correlated with cortical thinning in bilateral fronto-temporo-parietal regions. In mixed dementia patients, DR and DA changes across extensive WM regions correlated with cortical thinning in the bilateral fronto-temporo-parietal regions. Our findings showed that the relationships between GM and WM degeneration are distinct in pure ADD, pure SVaD, and mixed dementia, suggesting that different pathomechanisms underlie their correlations.

## Introduction

Alzheimer’s disease dementia (ADD) and subcortical vascular dementia (SVaD) are the two representative causes of dementia. ADD is characterized by amyloid plaques and neurofibrillary tangles accumulating in the gray matter (GM), which leads to neuronal death and cortical thinning^1^. SVaD is characterized by ischemic changes in the white matter (WM)^2^. However, it is well known that ADD patients also have WM microstructural changes^3–5^ and SVaD patients have cortical atrophy as well^6^.

The mechanism of WM-integrity disruption, along with GM degeneration (cortical thinning), in ADD and SVaD has not been well defined. In ADD patients, secondary Wallerian degeneration or WM degeneration independent of GM lesions has been suggested as a possible mechanism for WM microstructural changes^7, 8^. In SVaD, secondary axonal and trans-synaptic degeneration following subcortical injury or global ischemia in the cortex, as well as incidentally combined amyloid pathologies, have been proposed as the underlying mechanisms of cortical thinning. Despite these many hypotheses, there is a paucity of information on the correlation between WM and GM degeneration in ADD and SVaD patients.

To evaluate correlation between GM and WM degeneration, we first investigated the patterns of WM microstructural changes (using DTI) and GM degeneration (using cortical thickness analysis) in patients with pure ADD, pure SVaD, and mixed dementia, which were determined by Pittsburgh compound B (PiB) PET result and WM hyperintensities degree as described in the Methods. We have previously reported that both pure ADD and pure SVaD patients showed decreased FA and increased MD^5^. In this study, we further analyzed axial diffusivity (DA) and radial diffusivity (DR), which are diffusivities parallel or perpendicular to the WM fibers that provide more information regarding axonal or myelin damage, respectively^9, 10^. Then, we explored the correlation between WM microstructural changes and GM degeneration in each group using sparse canonical correlation analysis (sCCA), which makes it possible to combine different imaging modalities^11^. We hypothesized that in pure SVaD patients, changes in WM would be related to myelin breakdown and subsequent axonal damage. Thus, in pure SVaD, we expected an increase in DR and a decrease in FA to correlate with cortical thinning. On the other hand, we hypothesized that in pure ADD patients, cortical thinning would be the primary change leading to secondary Wallerian degeneration in adjacent WM. Thus, in pure ADD, we expected decreases in both DA and FA to correlate to cortical thinning^12^. Finally, we hypothesized that mixed dementia would show both ADD and SVaD characteristic and that an increase in DR and a decrease in DA would correlate to cortical thinning.

## Results

### Patient characteristics

Patient characteristics are shown in Table 1. PiB(+) SVaD patients were older than patients with PiB(+) ADD or PiB(−) SVaD. PiB(−) SVaD patients had higher MMSE scores than PiB(+) ADD and PiB(+) SVaD patients. However, there were no differences in Clinical Dementia Rating sum-of-boxes scores among the three groups of dementia patients.

**Table 1:** Subject demographics.

	NC (n = 56)	PiB(+) ADD (n = 40)	PiB(−) SVaD (n = 32)	PiB(+) SVaD (n = 23)
Age, years^a^	62.5 ± 7.5	67.0 ± 9.1*	71.3 ± 7.3*	78.1 ± 4.7*^†^^
Female, n (%)^b^	40 (71.4)	26 (65.0)	15 (46.9)	17 (73.9)
Education years^a^	12.5 ± 5.0	10.4 ± 5.3	8.6 ± 4.6*	9.1 ± 5.5*
WMH volume (cm^3^)^a^	0.72 ± 0.92	3.18 ± 6.04	39.29 ± 14.71^†^	45.54 ± 22.91^†^
Lacune, number^c^	0 (0, 0)	0 (0, 0)	13 (8.3, 33.3)^†^^	5 (2, 10)
Microbleed, number^c^	0 (0, 0)	0 (0, 0)	5.5 (1.0, 13.5)^†^	1 (0, 6)^†^
MMSE^a^	28.8 ± 1.4	17.4 ± 6.1*	21.6 ± 4.6*^†^^	18.7 ± 4.9*
CDR-SOB^a^	0.58 ± 0.45	4.85 ± 2.29*	5.97 ± 3.84*	6.54 ± 3.76*

Values are presented as ^a^mean ± *SD*, ^b^number of cases (percentage) or ^c^median (25^th^ percentile, 75^th^ percentile), *p < 0.05 compared to NC, ﻿^†^ *p* < 0.05 between PiB(+) ADD and PiB(−) SVaD or PiB(+) SVaD, ^*p* < 0.05 between PiB(−) SVaD and PiB(+) SVaD

Abbreviations: NC, normal controls; PiB, Pittsburgh compound B; ADD, Alzheimer’s disease dementia; SVaD, subcortical vascular dementia; SD, standard deviation; MMSE, Mini-Mental State Examination; CDR-SOB, Clinical Dementia Rating sum-of-boxes.

### White matter diffusivity properties in patients with PiB(+) ADD, PiB(−) SVaD, and PiB(+) SVaD

**Figure 1:**
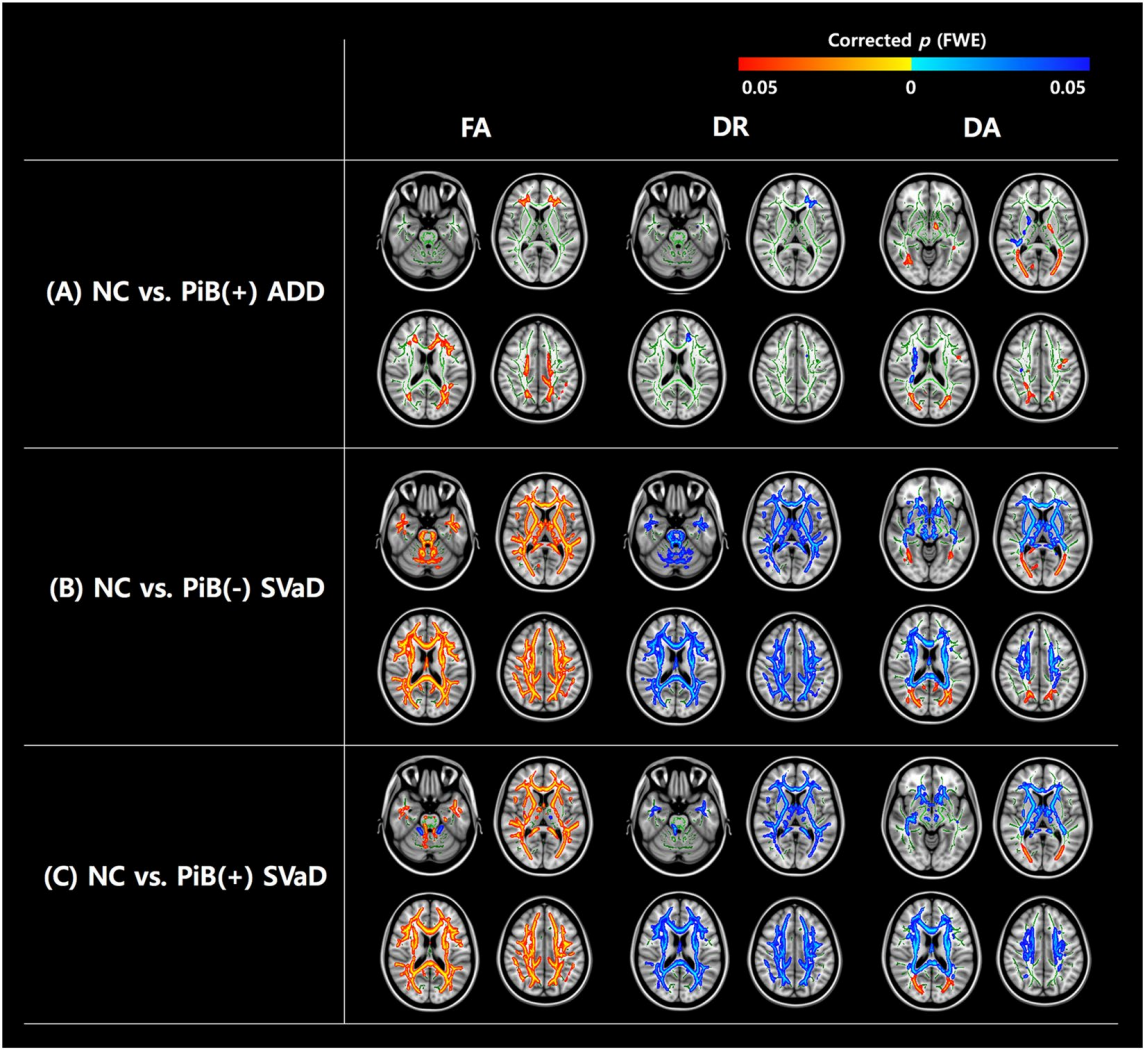
TBSS in patients with PiB(+) ADD, PiB(−) SVaD, and PiB(+) SVaD compared to normal controls (NC). Warm color (red-yellow) indicates lower DTI indices in the dementia group compared to the NC group while cold color (blue-light blue) indicates higher DTI indices in the dementia group compared to the NC group. These results are overlaid on the MNI152 standard brain and the mean skeleton image (green color, FA > 0.2). Abbreviations: FWE, family-wise error; FA, fractional anisotropy; DR, radial diffusivity; DA, axial diffusivity; NC, normal controls; PiB, Pittsburgh compound B; ADD, Alzheimer’s disease dementia; SVaD, subcortical vascular dementia; TBSS, tract-based spatial statistics; DTI, diffusion tensor imaging; MNI, Montreal Neurological Institute.

We compared WM microstructural changes of each dementia group to the NC group. FA changes in PiB(+) ADD and PiB(−) SVaD group compared to NC were reported in our previous study^5^ and thus, these results are not described here. The PiB(+) ADD group showed a focal higher DR in the left frontal WM region. DA was lower bilaterally in the parietal and occipital subcortical WM regions adjacent to the cortex, while it was higher in small areas including the corona radiata WM regions (Fig. 1A).

The PiB(−) SVaD group showed more extensive WM microstructural changes. DR was higher in all WM regions. DA was bilaterally higher in the centrum semiovale and corona radiata WM regions, while it was bilaterally lower in the parietal and occipital subcortical WM regions adjacent to the cortex (Fig. 1B).

WM microstructural changes of the PiB(+) SVaD group were similar to the PiB(−) SVaD group. FA was lower and DR was higher in all WM regions. DA was bilaterally higher in the centrum semiovale and corona radiata WM regions, while it was bilaterally lower in the parietal and occipital subcortical WM regions adjacent to the cortex (Fig. 1C). Details of the TBSS parameters are described in Table 2.

**Table 2:** Comparison of TBSS parameters.

Parameter	NC (n = 56)	PiB(+) ADD (n = 40)	PiB(−) SVaD(n = 32)	PiB(+) SVaD (n = 23)
FA	0.475 ± 0.016	0.473 ± 0.026	0.402 ± 0.032^*†^^	0.430 ± 0.026^*†^
DR (x10^−4^)	5.670 ± 0.268	5.833 ± 0.385	7.059 ± 0.653*^†^^	6.622 ± 0.540^*†^
DA (x10^−4^)	12.334 ± 0.269	12.298 ± 0.431	12.947 ± 0.470*^†^	12.874 ± 0.471^*†^

Values are means ± *SD*.

^*^ *p* < 0.05 compared to NC, ^†^ *p* < 0.05 between PiB(+) ADD and PiB(−) SVaD or PiB(+) SVaD, ^*p* < 0.05 between PiB(−) SVaD and PiB(+) SVaD. Abbreviations: TBSS, tract-based spatial statistics; NC, normal controls; PiB, Pittsburgh compound B; ADD, Alzheimer’s disease dementia; SVaD, subcortical vascular dementia; SD, standard deviation; FA, fractional anisotropy; DR, radial diffusivity; DA, axial diffusivity.

### Cortical thickness in patients with PiB(+) ADD, PiB(−) SVaD, and PiB(+) SVaD

**Figure 2:**
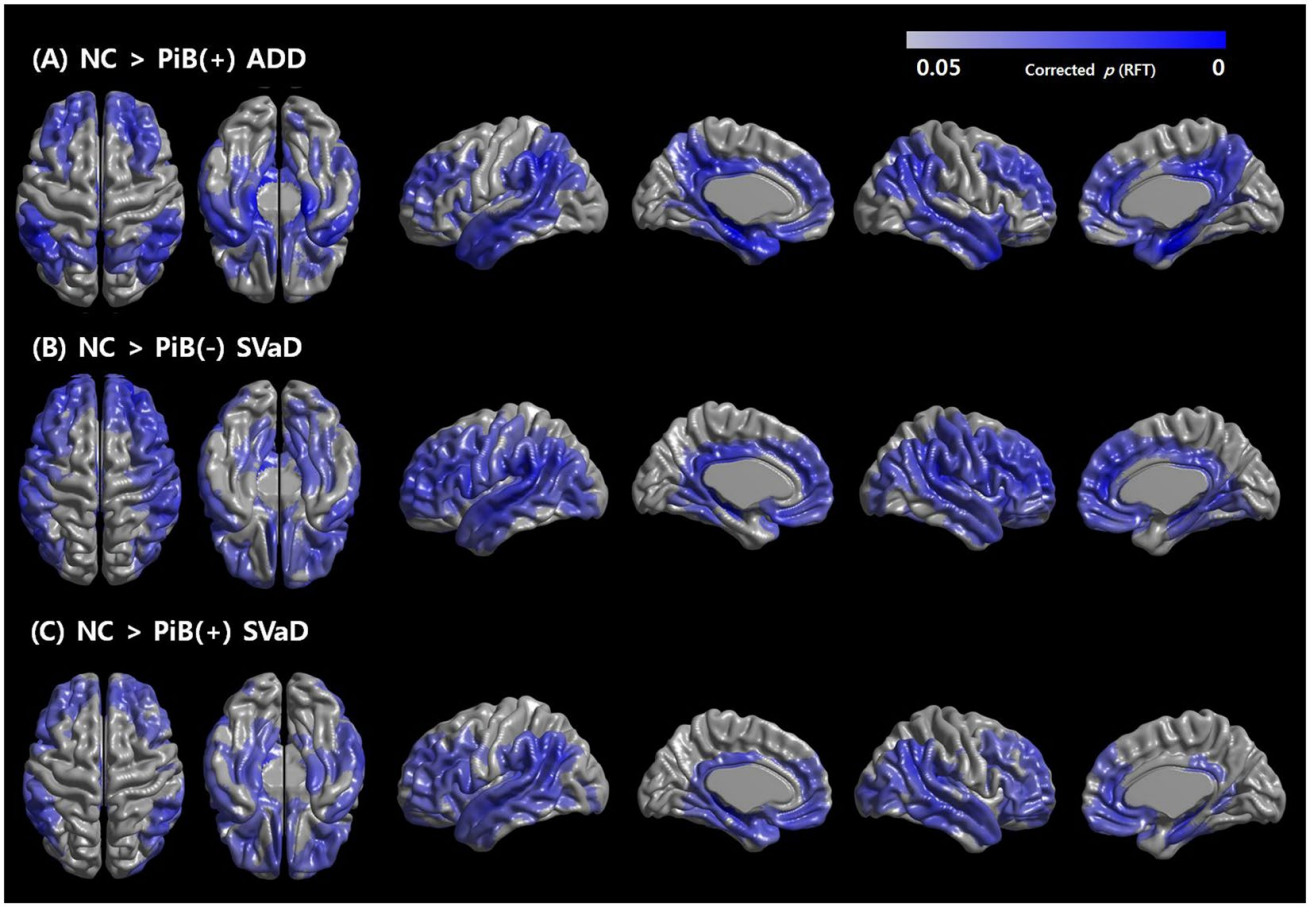
Topography or cortical thinning areas in patients with PiB(+) ADD, PiB(−) SVaD, and PiB(+) SVaD compared to NC. Abbreviations: RFT, random field theory; NC, normal controls; PiB, Pittsburgh compound B; ADD, Alzheimer’s disease dementia; SVaD, subcortical vascular dementia.

We compared cortical thickness of each dementia group to the NC group. The PiB(+) ADD group showed cortical thinning in diffuse areas including bilateral medial temporal and precuneus regions while the PiB(−) SVaD and the PiB(+) SVaD groups showed cortical thinning mainly in the frontal, inferior parietal, and temporal regions bilaterally, with medial temporal thinning more involved in the PiB(+) SVaD group (Fig. 2).

### Correlation between cortical thickness and white matter integrity

**Figure 3:**
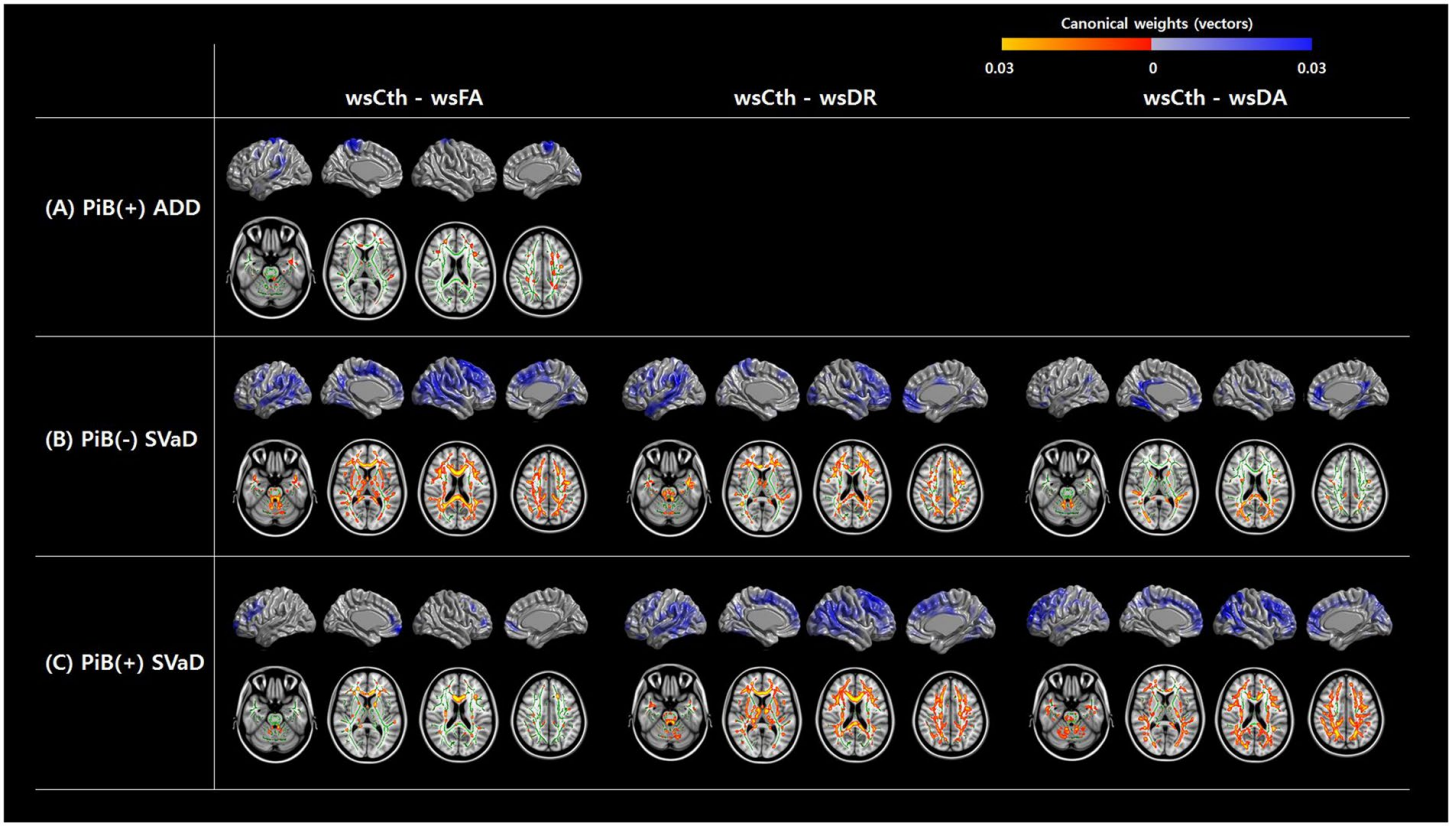
sCCA results showing regions of correlation between cortical thickness and DTI indices. A higher canonical weight number indicates higher correlation. Abbreviations: ws, W-score; Cth, cortical thickness; FA, fractional anisotropy; DR, radial diffusivity; DA, axial diffusivity; PiB, Pittsburgh compound B; ADD, Alzheimer’s disease dementia; SVaD, subcortical vascular dementia; sCCA, sparse canonical correlation analysis; DTI, diffusion tensor imaging.

In the PiB(+) ADD group, FA was the only DTI parameter that was correlated with cortical thickness. Variation of FA, limited to the frontal and parietal WM regions, correlated with variation of cortical thickness in the left inferior parietal lobule and bilateral paracentral lobules (Fig. 3A).

In the PiB(−) SVaD group, there were marked correlations between all DTI indices (especially FA) and cortical thickness. Variation of FA in extensive WM regions correlated with variation of cortical thickness in the bilateral temporo-parietal, medial frontal, and right frontal areas. Variation of DR in the frontal, temporal and parietal WM regions correlated with variation of cortical thickness in the bilateral lateral frontal, left lateral temporo-parietal, left paracentral lobule, and right medial frontal areas. Variation of DA in the bilateral parietal and occipital WM regions correlated with variation of cortical thickness in the inferior temporal, and bilateral anterior and posterior cingulate areas (Fig. 3B).

In the PiB(+) SVaD group, there were marked correlations between all DTI indices (especially DR and DA) and cortical thickness. Variation of FA in the anterior corpus callosum and scattered WM regions including centrum semiovale correlated with variation of cortical thickness in the bilateral frontal areas. Variation of DR in extensive WM regions correlated with variation of cortical thickness in the bilateral temporo-parietal, medial frontal, and right frontal areas. Variation of DA in extensive WM regions correlated with variation of cortical thickness in the bilateral frontal and right parietal areas (Fig. 3C).

## Discussion

We report a novel finding regarding correlation between WM microstructural changes and cortical thinning in pure ADD, pure SVaD, and mixed dementia. The first major finding is that in the pure ADD group, disruption of WM integrity was minimal with lower DA in WM adjacent to the cortex. In pure SVaD and mixed dementia, there was extensive disruption of WM integrity with higher DR and overall higher DA, but lower DA in WM adjacent to the cortex. The second major finding is that cortical thinning in pure SVaD strongly correlated with changes in FA and DR, while cortical thinning in mixed dementia strongly correlated with changes in DR and DA. Taken together, these findings suggest that the relationship between GM and WM degeneration differs according to the underlying pathobiology.

In pure ADD, TBSS revealed minimal disruption of WM integrity with lower DA in WM adjacent to the cortex, higher DA in small areas of deep WM, and higher DR in the left frontal WM area. Our finding that DA was especially lower in WM adjacent to the cortex in the pure ADD group is consistent with our hypothesis that Wallerian degeneration following neuronal death in the GM would damage axonal fibers leading to low DA. Meanwhile, we also found higher DA in small areas of deep WM regions as observed in previous studies^8, 13, 14^. This might be explained by complex pathobiologies: both axonal and myelin loss, subsequent increase in membrane permeability and glial alteration might cause water diffusion in unanticipated directions, leading to an increase in DA.

In pure SVaD and mixed dementia, TBSS showed extensive disruption of WM integrity with higher DR and DA. These results are consistent with previous studies showing that increases in DR and DA were associated with chronic WM degeneration^15, 16^. Interestingly, we found a unique pattern of DA change according to the distance from the cortex. DA was lower in the WM adjacent to the cortex in the posterior region, while it was higher in the deep WM regions, which might be related to different underlying pathobiologies. Although a similar pattern was found in the pure ADD group, DA was predominantly increased in the pure SVaD and mixed dementia groups. One possible explanation might be related to vulnerability to chronic ischemia. That is, in both pure SVaD and mixed dementia, deep WM regions, including periventricular WM areas, are more vulnerable to chronic ischemia which is represented by WMH on MRI^17^. Previous pathology studies showed that WMH regions had low myelin content and axonal loss or damage^18, 19^. This leads to a decrease in tract volume and an increase in the surrounding extracellular fluid, contributing to increased isotropic diffusivity with correspondingly higher DR and DA^16, 20^. On the other hand, WM regions adjacent to the cortex in pure SVaD and mixed dementia are relatively spared from chronic ischemia^17, 21^. Lower DA in this region may be due to secondary Wallerian degeneration from combined cortical atrophy^12^. Alternatively, the direction of DA change might reflect the stage of WM degeneration. A previous study showed that DA initially decreased after axonal damage and subsequently increased over time^16^. Therefore, higher DA in deep WM regions might be related to chronic WM degeneration, while lower DA adjacent to cortex might be related to recently developed WM degeneration.

sCCA revealed a disease-specific correlation pattern between GM and WM degeneration. Although the pure ADD group showed cortical thinning and significantly lower FA, there was little correlation between the two. In addition, no correlation was observed between lower DA (as determined by TBSS analysis) and cortical thinning. Although we may not have found significant correlations due to a small sample size, our findings suggest that the overall WM microstructural changes in pure ADD may occur independent of cortical pathology.

In the pure SVaD group, lower FA and higher DR (as determined by TBSS analysis) correlated with cortical thinning, while in the mixed dementia group, higher DR and higher DA (as determined by TBSS analysis) correlated with cortical thinning. In both the pure SVaD and mixed dementia groups, the cortical regions that correlated with WM integrity were mainly located in the lateral temporal, inferior parietal, and the lateral and medial frontal areas, which indicated that neurodegeneration in those areas paralleled WM degeneration. In both groups, chronic ischemia may primarily result in myelin breakdown, which is represented by higher DR. The degeneration process may propagate in a retrograde manner and result in neuronal cell death. Alternatively, disconnection of the cortical-subcortical loop may result in secondary cortical thinning. It is also possible that chronic ischemia may cause GM-WM correlation by simultaneously attacking GM and WM. However, the fact that different DTI indices correlated with cortical thinning in the pure SVaD and mixed dementia groups suggests that different pathomechanisms may underlie the GM-WM correlation in each disease. A higher vascular burden, such as lacunes, in pure SVaD might result in a more pronounced DR increase and FA decrease when compared to mixed dementia. In mixed dementia, the presence of an amyloid burden in addition to the chronic ischemic condition may alter the pattern of GM-WM correlation such that axial (DA) or radial (DR) diffusivity, rather than directionality of diffusion (FA), matters more for the association with widespread cortical thinning. Additionally, there was little correlation between lower FA and cortical thinning in mixed dementia patients, which suggested that these might occur independently in mixed dementia patients.

There are several limitations in this study. Firstly, we did not conduct PiB-PET scanning on the NC group. Because previous studies showed that approximately 10 to 30% of cognitively normal subjects are PiB positive^22, 23^, we might have underestimated the degree of WM microstructural changes and cortical thinning in the patient groups. Secondly, the mean ages of the NC and dementia groups were different. However, since the patients were consecutively recruited, we believe this reflected participant characteristics. To minimize the effect of age difference across the groups, we performed the analysis after adjusting for age. In addition, in the GM-WM correlation analysis, we calculated each patient’s W score, which is an age-adjusted Z score relative to the control group^24, 25^. Finally, sample size was relatively small and the number of subjects was not even across the four groups. Thus, we might have missed some important information because of low statistical power.

In conclusion, we revealed distinct GM-WM relationships in the dementia spectrum of ADD and SVaD (pure ADD, pure SVaD, and mixed dementia). We suggest that the relationship between GM and WM degeneration differs according to the underlying pathobiology of each condition.

## Methods

### Participants

We prospectively recruited 139 patients who were clinically diagnosed with SVaD (n = 70) or ADD (n = 69). Patients underwent ^11^C-PiB-PET scanning and MRI between September 2008 and August 2011. All SVaD patients fulfilled the Diagnostic and Statistical Manual of Mental Disorders - Fourth Edition (DSM-IV) criteria for vascular dementia and had severe WMH on MRI, which was defined as periventricular WMH (caps or band) ≥10 mm and deep WMH ≥25 mm in maximal diameter according to the modified Fazekas criteria^26^. Patients with WMH due to radiation injury, multiple sclerosis, vasculitis, or leukodystrophy (based on clinical history and other information such as blood test results) were excluded. Among the 70 SVaD patients (47 PiB(−) SVaD and 23 PiB(+) SVaD), 15 patients with PiB(−) SVaD were excluded due to preprocessing errors such as failure in brain mask extraction or registration to a common space.

ADD was diagnosed based on the criteria for probable AD proposed by the National Institute of Neurological and Communicative Disorders and Stroke-Alzheimer’s Disease and Related Disorders Association (NINDS-ADRDA)^27^. Among the 69 patients with ADD, we excluded 7 patients who were negative for PiB PET, 14 patients who exceeded mild WMH (periventricular WMH <10 mm and deep WMH <10 mm in maximal diameter), 2 patients who did not have one of the required imaging modalities (DTI or FLAIR), and 6 patients who showed preprocessing errors such as failure in brain mask extraction or registration to a common space. We also recruited 56 normal controls (NC) who had no history of neurologic or psychiatric illnesses and no abnormalities detected during neurological examination. These were classified to be cognitively normal by neuropsychological testing. All the NC had no or mild WMH (periventricular WMH <10 mm and deep WMH <10 mm in maximal diameter) on MRI.

The final patient sample consisted of 32 patients with PiB(−) SVaD (pure SVaD), 23 patients with PiB(+) SVaD (mixed dementia), 40 patients with PiB(+) ADD (pure ADD), and 56 NC. The same study sample of pure SVaD, pure ADD, and NC was used in our previous study^5^. The criteria for a PiB-positive scan are described in the *PET acquisition and data analysis* section.

We obtained written informed consent from each participant and the Institutional Review Board of Samsung Medical Center approved the study protocol. All methods were carried out in accordance with the approved guidelines.

### MRI techniques

Patients underwent standardized T2, three-dimensional (3D) T1 turbo field echo, 3D fluid-attenuated inversion recovery (FLAIR), and DTI images at Samsung Medical Center using a 3.0T MRI scanner (Philips 3.0T Achieva). For 3D T1 turbo field echo MR images, the following parameters were used: sagittal slice thickness, 1.0 mm, over contiguous slices with 50% overlap; no gap; repetition time (TR) of 9.9 msec; echo time (TE) of 4.6 msec; flip angle of 8°; and matrix size of 240 × 240 pixels, reconstructed to 480 × 480 over a field of view (FOV) of 240 mm. For 3D FLAIR images, we used the following parameters: axial slice thickness of 2 mm; no gap; TR of 11000 msec; TE of 125 msec; flip angle of 90°; and matrix size of 512 × 512 pixels. For whole-brain DT-MRI examinations, sets of axial diffusion-weighted single-shot echo-planar images were obtained: 128 × 128 acquisition matrix, 1.72 × 1.72 × 2 mm^3^ voxels; reconstructed to 1.72 × 1.72 × 2 mm^3^; 70 axial slices; 22 × 22 cm^2^ field of view; TE 60 ms, TR 7696 ms; flip angle 90°; slice gap 0 mm; b-factor of 600 smm^−2^. We acquired diffusion-weighted images from 45 different directions. We used the baseline image without weighting [0, 0, 0].

### PET acquisition and data analysis

All patients completed a ^11^C-PiB PET scan at Samsung Medical Center or Asan Medical Center with identical settings using a Discovery STe PET/CT scanner (GE Medical Systems, Milwaukee, WI, USA). To measure PiB retention, we used the cerebellar gray matter as the reference region and calculated the cerebral cortical region to cerebellum uptake ratio. Patients were considered PiB-positive if their global PiB uptake ratio was more than two standard deviations from the mean of the normal controls (PiB retention ratio ≥1.5)^28^. The detailed radio chemistry profiles, scanning protocol and PiB-PET data analysis are described in the supplementary information.

### DTI Processing

FMRIB’s Software Library (FSL v5.0.2.1, http://www.fmrib.ox.ac.uk/fsl) was used to process DTI data. We corrected motion artifacts and eddy current distortions by normalizing each diffusion-weighted volume to the non-diffusion-weighted volume (b0) by applying the affine registration method in the FMRIB’s Linear Image Registration Tool (FLIRT v6.0). Using a general linear-fitting algorithm, diffusion tensor matrices were generated from the sets of diffusion weighted images. Subsequently, we obtained FA, DA, and DR for each voxel according to simple linear fitting algorithm using the DTIFIT Tool (part of FSL).

### Tract-based spatial statistics (TBSS) analysis

The FA, DA and DR maps of DTI pre-processing results were used for TBSS analysis^29^. We aligned all FA images onto a standard FMRIB58 FA template which was provided by the FSL software. In this process, we used a nonlinear registration algorithm implemented in the TBSS package. After alignment, the FA images were averaged to create a skeletonized mean FA image. Aligned FA images from each patient were projected onto the skeleton by filling the skeleton with the highest FA values at the nearest relevant center of fiber tracts. We chose a threshold FA value of 0.2 to exclude voxels of GM or cerebrospinal fluid (CSF). DR and DA images were also processed by implementing the FA nonlinear registration and projecting them onto the skeleton using identical methods to those derived from the original FA data.

### Image processing for cortical thickness measurement

The CIVET anatomical pipeline was used to extract cortical thickness (http://mcin-cnim.ca/neuroimagingtechnologies/civet/)^30^. In brief, using a linear transformation, native MRI images were registered to the Montreal Neurological Institute (MNI) 152 standard space^31^. The N3 algorithm was used to correct the images for intensity-based non-uniformities^32^ caused by non-homogeneities in the magnetic field. Then, the registered and corrected images were classified into WM, GM, CSF, and background using a 3D stereotaxic brain mask and the Intensity-Normalized Stereotaxic Environment for Classification of Tissues (INSECT) algorithm^33^. The surfaces of the inner and outer cortex were automatically extracted using the Constrained Laplacian-based Automated Segmentation with Proximities (CLASP) algorithm^34^.

Cortical thickness was defined as the Euclidean distance between the linked vertices of the inner and outer surfaces; there were 40,962 vertices in each hemisphere in native space^34^. The thickness value was spatially normalized using surface-based two-dimensional registration with a sphere-to-sphere warping algorithm. Thus, the vertices of each subject were nonlinearly registered to a standard surface template to compare thickness across subjects^35, 36^. Cortical thickness was subsequently smoothed using a surface-based diffusion kernel in order to increase the signal-to-noise ratio. We chose a 20-mm full-width at half-maximum kernel size to maximize statistical power while minimizing false positives^37^.

The presence of extensive WMH in the MRI scans made it difficult to completely delineate the inner cortical surface with the correct topology due to tissue classification errors. To overcome this technical limitation, we automatically defined the WMH region using a FLAIR image and substituted it for the intensity of peripheral, normal-appearing tissue on the high-resolution T1 image after affine co-registration, as described in earlier studies^38^. This method was applied to all participants.

### W-score maps

We carried out W-score mapping to identify the degree of cortical atrophy and WM microstructural changes in each patient using cortical thickness and DTI measures (i.e., FA, DA and DR) with the NC as a reference. Details on the theory and computation of W-scores are available elsewhere^25, 39^. In this study, W-score maps were computed vertex-wise for the surface model and voxel-wise for the skeletonized volume of each imaging data set according to the following formula: W-score = [(patient’s raw value) − (value expected in the control group for the patient’s age, sex, and level of education)]/(SD of the residuals in the control group). W-scores are similar to Z-scores in that they have a mean value of 0 and a SD of 1 in the control group, and values of +1.65 and −1.65 correspond to the 95^th^ and 5^th^ percentiles, respectively. However, W-scores are adjusted for specific covariances such as age, sex, and level of education. To avoid confusion with respect to W-score direction in cortical thickness and DTI measures, we used the W-scores as positive values indicating larger cortical thickness and larger values of FA, DA and DR.

### Sparse canonical correlation analysis (sCCA)

Because traditional canonical correlation analysis is severely limited when the dimensionality of the data is larger than the number of subjects^11^, sCCA was used^40–43^. sCCA attempts to find relationships between two sets of multi-voxel or multi-vertex dimensional W-scores of DTI measures (i.e., FA,DA, DR) and cortical thickness independently obtained from DTI and T1 imaging modalities. In other words, it simultaneously finds the canonical weight vectors in each imaging modality that maximize the correlation of the projections of each DTI measure and cortical thickness input data onto their canonical weight vectors. sCCA is particularly useful in that it controls the influence of outliers on the computed correlations and automatically locates the most reliable and informative sets in one modality that are related to another modality using a sparse selection process with a regularized energy-minimization approach. sCCA for each patient group was carried out using R packages (http://cran.r-project.org/web/packages/PMA/) with positivity and sparseness constraints. The sparseness parameters, which control the sparsity for either set of the canonical variates, were selected as approximately half of the voxel/vertex dimension of the input data to focus on spatially-distributed patterns. For visualization, canonical weight vectors of each imaging data set were mapped onto an ICBM 152 surface model for cortical thickness and volume template for DTI measures. We used permutation testing of 2,000 iterations to assess sCCA significance. For permutation testing, we randomly reordered the possible pairs of the two input images (one of the DTI measures (i.e., FA, DA, DR) and the cortical thickness). However, the two images were not selected from the same subject. The p-value for canonical variates was estimated as the ratio of the number of the permutations in which correlation value exceeded the original correlation value to the number of total permutations. Significance was defined as p < 0.05.

### Statistical analysis

To analyze demographic and patient characteristic data, one-way ANOVA for continuous variables and Chi-square tests for dichotomous variables were performed. A two-sided *p* < 0.05 was considered statistically significant.

To test for localized differences in the DTI measures of each dementia group compared with the NC group, voxel-wise statistical analysis of individual skeleton images was performed using a nonparametric permutation test. We included age, sex, and level of education as covariates in the analysis of covariance (ANCOVA), and the null distribution was built up over 5,000 permutations. We used threshold-free cluster-enhancement (TFCE) with the 2D parameter settings^44^ to avoid an arbitrary threshold of an initial cluster-formation. To correct for multiple comparisons, the results for DTI measures (i.e., FA, DA and DR) were considered significant at a family-wise error (FWE)-corrected P < 0.05. To estimate the differences in cortical thickness for each dementia group compared with the NC group, general linear modeling and random field theory (RFT) were applied using the SurfStat toolbox (http://www.math.mcgill.ca/keith/surfstat/)^45^. General linear analysis was performed after controlling for age, sex, and level of education. To correct for multiple comparisons, the results for cortical thickness were considered to be significant at RFT corrected p < 0.05.

The results of cortical thickness and DTI measures were projected on an ICBM 152 surface template and volume template for visualization.

## Electronic supplementary material

Supplementary information
