# Supplementary material for: Correlations between Gray Matter and White Matter Degeneration in Pure Alzheimer’s Disease, Pure Subcortical Vascular Dementia, and Mixed Dementia
Source: Sci Rep. 2017 Aug 25;7:9541. doi: 10.1038/s41598-017-10074-x (PMC5573310; doi:10.1038/s41598-017-10074-x)
Supplement: Supplementary file 1 — Supplementary information [file 41598_2017_10074_MOESM1_ESM.doc]

**Correlations between Gray Matter and White Matter Degeneration in Pure Alzheimer’s Disease, Pure Subcortical Vascular Dementia, and Mixed Dementia**

Hyemin Jang^a,d*^, Hunki Kwon^e*^, Jin-Ju Yang^e^, Jinwoo Hong^e^, Yeshin Kim^a,d^, Ko Woon Kim^a,d^, Jin San Lee^f^, Young Kyoung Jang^a,d^, Sung Tae Kim^b^, Kyung Han Lee^c^, Jae Hong Lee^g^, Duk L. Na^a,d,h,i^, Sang Won Seo^a,d,h,j^, Hee Jin Kim^a,d†^, Jong-Min Lee^e†^

^*^These authors contributed equally to this work

^†^These authors contributed equally to this work

^a^Department of Neurology,^b^Radiology and ^c^Nuclear Medicine, Samsung Medical Center, Sungkyunkwan University School of Medicine, Seoul, Korea

^d^Neuroscience Center, Samsung Medical Center, Seoul, Korea

^e^Department of Biomedical Engineering, Hanyang University, Seoul, Korea

^f^Department of Neurology, Kyung Hee University Hospital, Seoul, Korea

^g^Department of Neurology, Asan Medical Center, Ulsan University School of Medicine, Seoul, Korea

^h^Department of Health Sciences and Technology, SAIHST, Sungkyunkwan University, Seoul, Korea

^i^Stem Cell & Regenerative Medicine Institute, Samsung Medical Center, Seoul, Korea

^j^Department of Clinical Research Design & Evaluation, SAIHST, Sungkyunkwan University, Seoul, Korea

**PiB-PET acquisition**

[^11^C] PiB-PET scanning was performed in 3-dimensional scanning mode that examined 35 slices of 4.25-mm thickness spanning the entire brain. [^11^C] PiB was injected into an antecubital vein as a bolus with a mean dose of 420 MBq (i.e., range 259–550 MBq). A CT scan was performed for attenuation correction 60 minutes after injection. A 30-minute emission static PET scan was then initiated. The specific radioactivity of [11C] PiB at the time of administration was more than 1,500 Ci/mmol for patients and the radiochemical yield was more than 35%. The radiochemical purity of the tracer was more than 95% in all PET studies.

**PiB-PET data analysis**

PiB PET images were co-registered to individual MRIs, which were normalized to a T1-weighted MRI template. Using these parameters, MRI co-registered PiB PET images were normalized to the MRI template. The quantitative regional values of PiB retention on the spatially normalized PiB images were obtained by an automated VOIs analysis using the automated anatomical labeling (AAL) atlas. Data processing was performed using SPM Version 5 (SPM5) within Matlab 6.5 (MathWorks, Natick, MA).

We selected 28 cortical VOIs from left and right hemispheres using the AAL atlas. The cerebral cortical VOIs that were chosen for this study consisted of the bilateral frontal (superior and middle frontal gyri, the medial portion of superior frontal gyrus, the opercular portion of inferior frontal gyrus, the triangular portion of inferior frontal gyrus, supplementary motor area, orbital portion of the superior, middle, and inferior orbital frontal gyri, rectus and olfactory cortex), posterior cingulate gyri, parietal (superior and inferior parietal, supramarginal and angular gyri, and precuneus), lateral temporal (superior, middle and inferior temporal gyri, and heschl gyri), and occipital (superior, middle, and inferior occipital gyri, cuneus, calcarine fissure, and lingual and fusiform gyri). Regional cerebral cortical uptake ratios were calculated by dividing each cortical VOI’s uptake ratio by the mean uptake of the cerebellar cortex (cerebellum crus1 and crus2). Global PiB uptake ratio was calculated from the volume-weighted average uptake ratio of bilateral 28 cerebral cortical VOIs.
